# Low-Temperature Synthesis of Bi_2_S_3_ Hierarchical Microstructures via Co-Precipitation and Digestive Process in Aqueous Medium

**DOI:** 10.3390/ma17081818

**Published:** 2024-04-16

**Authors:** José Alfonso Carrasco-González, Rebeca Ortega-Amaya, Esteban Díaz-Torres, Manuel A. Pérez-Guzmán, Mauricio Ortega-López

**Affiliations:** 1 Sección de Electrónica del Estado Sólido, Departamento de Ingeniería Eléctrica, Centro de Investigación y de Estudios Avanzados del Instituto Politécnico Nacional, Av. IPN No. 2508, Ciudad de México 07360, Mexico; jose.carrasco@cinvestav.mx (J.A.C.-G.); ediaz@cinvestav.mx (E.D.-T.); 2 Programa de Doctorado Transdisciplinario en Desarrollo Científico y Tecnológico para la Sociedad, Centro de Investigación y de Estudios Avanzados del Instituto Politécnico Nacional, Av. IPN No. 2508, Ciudad de México 07360, Mexico; 3 CICFIM-Facultad de Ciencias Físico Matemáticas, Universidad Autónoma de Nuevo León, Av. Universidad S/N, Cuidad Universitaria, San Nicolás de los Garza, Nuevo León 66451, Mexico; orebeca@ymail.com; 4 Departamento de Física, Universidad Autónoma Metropolitana-Iztapalapa, Av. San Rafael Atlixco No. 186, Ciudad de México 09340, Mexico

**Keywords:** Bi_2_S_3_, co-precipitation, hierarchical self-assembly, microstructure

## Abstract

Bismuth sulfide (Bi_2_S_3_) nanostructures have gained significant attention in the fields of catalysis, optoelectronics, and biomedicine due to their unique physicochemical properties. This paper introduces a simple and cost-effective method for producing Bi_2_S_3_ microstructures at low temperatures (25 and 70 °C). These microstructures are formed by the hierarchical self-assembly of Bi_2_S_3_ nanoparticles, which are typically 15–40 nm in size. The nanoparticles are synthesized by the co-precipitation of thioglycolic acid, thioacetamide, and bismuth nitrate in water. The study delves into the phase composition and morphological evolution of the microstructures, concerning the chemical composition of the solution and the synthesis temperature. X-ray analysis has confirmed the formation of single-phase bismuthinite Bi_2_S_3_. The synthesis process generates primary building blocks in the form of 15–40 nm Bi_2_S_3_ nanocrystals, which then go through a hierarchical self-assembly process to produce a range of micrometer-sized structures. A scanning electron microscopy examination revealed that the primary nanoparticles self-assemble into quasi-1D worm-like nanostructures, which then self-assemble to create sponge-shaped microstructures. These structures subsequently self-organize and refine into either flower- or dandelion-like microstructures, mostly depending on the synthesis temperature and the chemistry of the digestion medium.

## 1. Introduction

The development of advanced materials with unique properties holds immense potential for various technological and scientific applications. Amongst these, 3D hierarchical nanostructures of binary V-VI compounds have gained significant attention due to their exceptional structural architecture and enhanced performance in energy conversion-related and environmental remediation applications [1]. Therefore, it is crucial to establish cost-effective and straightforward processes for synthesizing these materials. In this regard, colloidal chemistry in an aqueous medium can play a pivotal role in achieving this objective.

The semiconductor material bismuth sulfide (Bi_2_S_3_) has raised special interest due to its optical and electrical properties, which can be widely tailored by controlling the size and shape of its morphological features during synthesis [2,3,4,5,6]. Crystalline Bi_2_S_3_ exhibits n-type conductivity and has a direct band with a band gap energy of E_g_ = 1.4 eV. However, various synthesis methods for Bi_2_S_3_ nanocrystals reported E_g_ values ranging from 1.3 to 1.7 eV [6,7]. In comparison to other highly toxic mineral sulfides such as Greenockite (CdS), Orpiment (As_2_S_3_), or Cinnabar (HgS), Bismutinite (Bi_2_S_3_) can be considered a compound with low or no toxicity [8,9,10,11].

Bismuth sulfide exhibits a unique crystal structure, characterized by a unit cell that belongs to the orthorhombic crystalline system Pnma. The lattice parameters for Bi_2_S_3_ are *a* = 11.15 Å, *b* = 11.30 Å, and *c* = 3.981 Å. Within its unit cell, there are 20 atoms, which consist of four Bi_2_S_3_ units. These atoms arrange themselves in slats along the *c*-axis and are stacked together by intermolecular bonds along the *a*-axis, resulting in the formation of three-dimensional lamella-like structures [12]. These structural properties allow Bi_2_S_3_ to develop crystalline habits such as nanorods, nanosheets, and acicular structures [13,14,15,16,17,18,19,20,21].

Recently, nanostructured Bi_2_S_3_ has attracted significant attention due to its ease of preparation using various techniques. Chemical methods where a powdered material [22], thin films [23], and solutions [24] were obtained and physical methods that resulted in films and bulk materials [25,26,27] were proven. Consequently, this has allowed extensive bismuth sulfide study of its potential applications in polymer/Bi_2_S_3_ hybrid solar cells, hydrogen storage [28,29], and thin films for electronic devices [30,31,32].

The primary aim of this study is to synthesize nanostructures of Bi_2_S_3_ utilizing a cost-effective and straightforward process, such as co-precipitation in an aqueous medium.

## 2. Experimental Procedures

### 2.1. Reagents and Material Synthesis

The Bi_2_S_3_ nanostructures were prepared by co-precipitation using aqueous solutions of bismuth nitrate pentahydrate (Bi(NO_3_)_3_•5H_2_O, ≥98% Sigma-Aldric, Toluca, Mexico), thioglycolic acid (TGA, HSCH_2_CO_2_H, ≥98% Sigma-Aldrich), and thioacetamide (TAA, H_2_C=CSNH_2_, Analytyka, Nuevo Leon, Mexico). All reagents are used without any additional purification process.

In all experiments, the precursor solutions of sulfur and bismuth ions were separately prepared as follows:

Solution A. The TAA solution was prepared at room temperature by dissolving 451 mg of TAA in 60 mL of deionized water for 3 min, with the concentration being 0.1 M in TAA. 

Solution B: The bismuth nitrate solution involved the direct dissolution of the bismuth salt in TGA within a ball flask with constant magnetic stirring. After complete dissolution (~7 min), 5 mL of deionized water was added, and it was either heated up to 70 °C or maintained at 25 °C for 15 min. Subsequently, 30 mL of solution A was mixed with 30 mL of deionized water. The resulting solution was completely mixed with solution B. This mixing triggered a rapid reaction, causing the solution color to change from yellow to orange, then red, and finally a dark brown. The colloidal reaction was allowed to proceed for 60 min at the chosen synthesis temperature. The minimum reported reaction time is that measured just after the mixing process was performed, being around 3 min.

**Figure 1:**
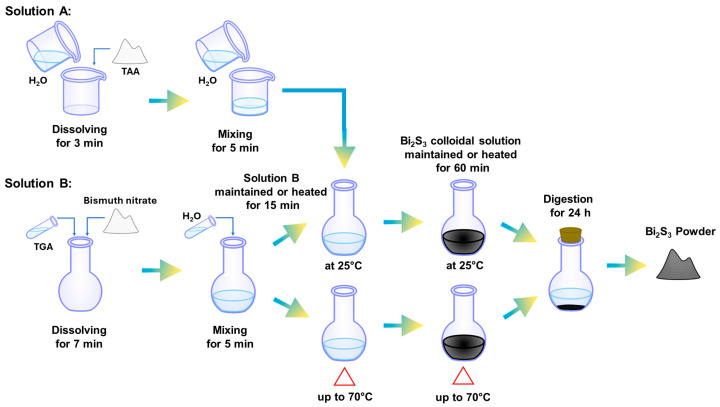
Schematic representation of Bi_2_S_3_ colloidal synthesis process, indicating the dissolving, mixing, reaction, digestion, and powder obtention stages.

Following the synthesis process, the heating source was turned off, and the resulting solution was left to undergo a 24 h digestion period. This digestion process facilitates the separation of the solid phase, forming a sedimented floc, from the liquid phase. The liquid phase, containing dispersed Bi_2_S_3_ nanoparticles and byproducts, was carefully poured off. To ensure a neutral pH, the floc was subjected to multiple washes. Finally, the floc was effectively dried by heating it at 70 °C. Figure 1 details the entire Bi_2_S_3_ synthesis process described in this section.

Four series of experiments were prepared and labeled as BiS10X, BiS20X, and BiS30X (X = 1–3), where the TAA concentration, the TGA one, and the Bi(NO_3_)_3_•5H_2_O one were varied, respectively, and the BiSTAX series, where the reaction was performed at 25 °C, varying the TGA concentration. However, while some experiments produced samples with similar structural and morphological features, the most notable distinction was observed in samples prepared at varying temperatures. For the purpose of our discussion, we have selected samples prepared at 25 and 70 °C as representative examples.

### 2.2. Characterization Techniques

The phase composition of Bi_2_S_3_ nanostructures was assessed by X-ray diffraction (XRD), and a PANalytical X’Pert-PRO diffractometer (Malvern Panalytical, Malvern, UK) with Cu-Kα emission was used for this purpose. The scan range was set from 15° to 70° with a step size of 0.04°. The crystal size was determined by analyzing the position of the highest intensity peaks and their corresponding Full Width at Half Maximum (FWHM) using the Sherrer equation, with a value of *k* = 0.9 [33]. The morphological characterization of the powders was carried out using scanning electron microscopy (SEM), where a FE-SEM Zeiss Auriga (Carl Zeiss Microscopy GmbH, Jena, Germany) operating at 5 and 10 kV was utilized for this analysis.

## 3. Results and Discussion

The phase composition and morphology of the Bi_2_S_3_ powder were analyzed by XRD and SEM, respectively. As stated above, various experimental conditions led to samples with similar compositions and morphological developments. The following discussion is based on the representative samples synthesized at room temperature (~25 °C, BiSTAX) and 70 °C (BiS10X). 

### 3.1. XRD Analysis

**Figure 2:**
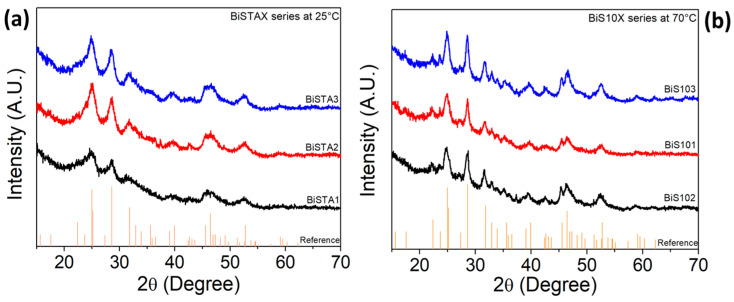
XRD patterns of the Bi_2_S_3_ samples. (**a**) Samples corresponding to the BiSTAX series (synthesized at 25 °C) and (**b**) samples corresponding to the BiS10X series (synthesized at 70 °C). Notice that the vertical lines belonging to the bismuthinite mineral reference card ICCD No. 00-006-0333 were added.

The diffractograms shown in Figure 2 correspond to the Bi_2_S_3_ samples corresponding to the BiSTAX series (Figure 2a) and BiS10X series (Figure 2b). It is worth noting that well-crystallized samples were obtained, despite being prepared at low temperature (25 °C). All the observed diffraction lines are attributed to the orthorhombic phase of Bi_2_S_3_ bismuthinite, according to the ICCD No. 00-006-0333 reference card. No diffraction peaks corresponding to solid phases other than bismuthinite Bi_2_S_3_ were detected. As previously mentioned, the Bi salt was directly mixed with the TGA to minimize Bi-ion hydrolysis and the subsequent formation of complex hydrated bismuth oxide species [29]. This is because both thioglycolic acid and thioacetamide serve a dual role as Bi-ion ligands and as a source of sulfur (S) [30,31]. This successful process of preparing the precursor solution effectively prevents the formation of impurity phases other than Bi_2_S_3_.

The lattice parameters were estimated using the XRD data from the most intense peaks, resulting around *a* = 11.18 Å, *b* = 11.43 Å, and *c* = 3.98 Å. These values slightly differ from those reported in the ICCD reference card. That is, *a* and *b* are larger than the reference values in about 0.27 and 1.15%, respectively, indicating a tensile stress; on the contrary, the lattice parameter *c* resulted in a minimum compression stress of 0.03%, which represents a practically negligible difference. The crystallite size, on the other hand, was found to vary in the 15–40 nm range. Nevertheless, as discussed below, in some cases, certain experimental parameters slightly affected the final morphology of the self-assembled microstructures.

### 3.2. Morphological Analysis

**Figure 3:**
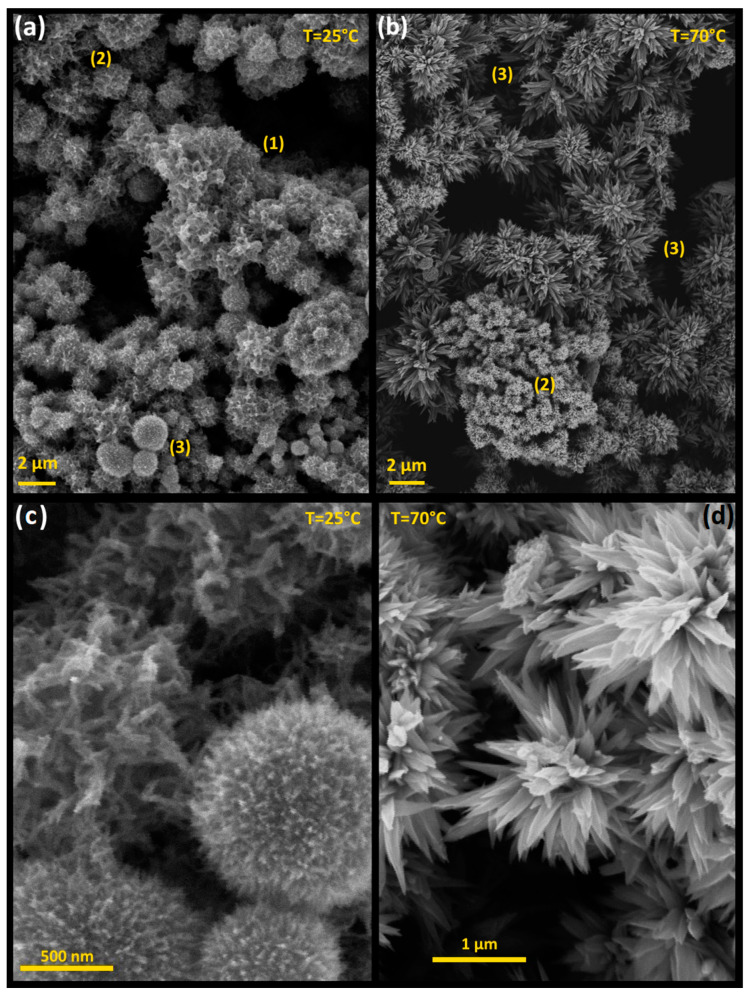
(**a**,**b**) Panoramic SEM images of the two Bi_2_S_3_ crystalline acicular structures, simultaneously displaying different self-assembly stages. (1) indicates the early stage (worm formation), (2) sponge-like formation stage, and (3) formed acicular structures (dandelions at 25 °C or flowers at 70 °C). (**c**,**d**) Close-up view of dandelion and flower Bi_2_S_3_ crystalline microstructures.

The morphology of the Bi_2_S_3_ powder was analyzed using SEM. Figure 3 presents SEM images of representative samples prepared at room temperature (25 °C) (Figure 3a) and 70 °C (Figure 3b). The synthesis procedure, which combines co-precipitation and digestion, resulted in Bi_2_S_3_ microstructures with a wide variety of forms, all seemingly derived from a self-assembly process. The XRD analysis confirmed that these microstructures are composed of 15–40 nm-in-size Bi_2_S_3_ nanoparticles, whereas the SEM images revealed larger Bi_2_S_3_ structures in the micrometer range. Therefore, the SEM characterization suggests that the synthesis procedure produces nanosized Bi_2_S_3_ as the primary building blocks, from which microstructures develop through sequential self-assembly steps. The dominant morphologies that emerged at different temperatures are highlighted in Figure 3. At room temperature, Figure 3a,c show the formation of sponge-, urchin-, and dandelion-like bismuth sulfide microstructures, which were influenced by the TGA content. On the other hand, at 70 °C (Figure 3b,d), sponge-, urchin, flower- and coral-like microstructures are predominantly observed under all the tested experimental conditions. Detailed close-up views of each morphology can be seen in Figure 3c,d. The sponge-like and dandelion structures have average sizes of 1.16 µm and 0.90 µm, respectively, while the flower-like structure is 1.30 µm in size, and the maximal visible cross-sectional length of their leaves is 122.73 nm.

**Figure 4:**
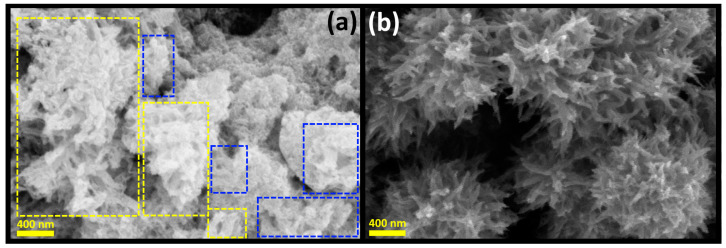
SEM images of Bi_2_S_3_ crystals in some stages of the self-assembly process. (**a**) Rolled-up worm formation and (**b**) sponge-like structure formation stage. Rectangles were added to the image (**a**) to indicate the unrolled (blue) and rolled-up worm (yellow) crystalline structures.

To gain a better understanding of the sequential self-assembly process, aliquots from representative precipitation reactions were taken out ~1 min after starting the precipitation reaction. Figure 4a illustrates the morphological evolution of Bi_2_S_3_ microstructures after ~1 min of initiating phase solid precipitation at 70 °C. It is evident that upon the onset of precipitation, nanoscale Bi_2_S_3_ particles undergo self-assembly to form worm-like nanostructures, which then further assemble into intricate sponge-like microstructures, indicating that the hierarchical self-assembly of Bi_2_S_3_ nanoparticles begins accompanying the solid phase precipitation and continues to progress during the digestive process, as shown in Figure 3b. Figure 4a reveals that worm-like structures are commonly observed in samples synthesized at both 25 °C and 70 °C and that they self-assemble into a woven network sponge-like microstructure, as shown in Figure 4b.

We have proposed a potential pathway that leads to the formation of microstructures by closely examining the SEM images shown in Figure 3a,b. We have assumed that these figures serve to illustrate the critical steps of the self-assembly process. That is, we propose that the SEM images include micron-sized Bi_2_S_3_ with different degrees of development. The different stages of self-assembly and formation are highlighted in Figure 3a,b, denoted by numbers, illustrating (1) the early stage of worm formation, (2) the sponge-like formation stage, and (3) the final stage of acicular microstructure formation. Based on Figure 3a, we propose that the morphology progresses sequentially as follows:

Initially, primary Bi_2_S_3_ nanoparticles self-assemble into worm-like 1D nanostructures, which subsequently group and coalesce into waved worm-like structures resembling sponge-like microstructures. The subsequent shape modification of self-assembled Bi_2_S_3_ microstructures develops during the digestion process. Figure 4a illustrates the morphological details of the Bi_2_S_3_ microstructures. It is evident that at the early stage of the self-assembly process, worm- and sponge-like microstructures emerged as the dominant ones. These develop into porous Bi_2_S_3_ microstructures resembling a sponge-like structure, which are prone to further shape transformations during the digestion process, depending on the temperature. The worm-like and sponge-shaped structures appear to be a common occurrence across all the samples, regardless of the experimental conditions used for their preparation. This assertion is illustrated by Figure 4b for synthesis at room temperature.

Following this starting formation, the sponge-shaped microstructures are subjected to Ostwald ripening, redissolution, and reshaping during digestion to achieve their final shape. At 25 °C, the mechanisms responsible for consolidating and refining the crystalline microstructure typically result in mostly porous, dandelion-like Bi_2_S_3_ structures, as seen in Figure 3a,c. Conversely, the samples prepared at 70 °C exhibit a transformation from nanoworms into acicular structures, which then self-assemble to form flower-like microstructures, as shown in Figure 3b,d.

**Figure 5:**
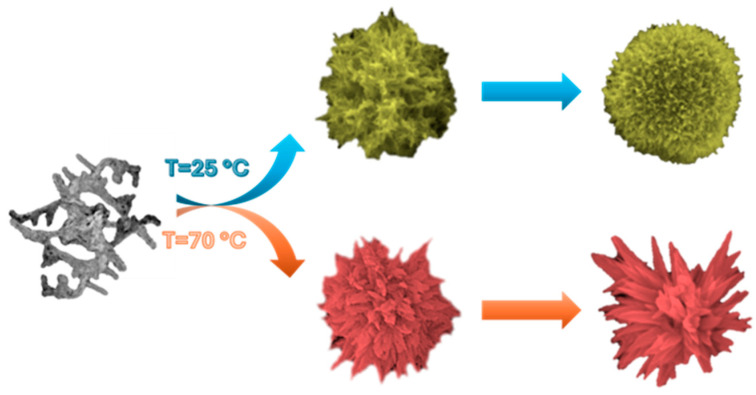
Schematic representation of the hierarchical self-assembly process of Bi_2_S_3_ crystals changing into sponge-like and acicular structures.

Figure 5 provides a summary of the self-assembly and digestion processes described above.

To compare our results with other related works, it is worth mentioning that the synthesis of bismuth sulfide microstructures has extensively been studied, and Bi_2_S_3_ microstructures like those reported here have been observed using both chemical and physical synthesis. For instance, urchin-like Bi_2_S_3_ nanocrystals were obtained by Ma, Li, Chen, Sasikala, and Sang [21,22,34,35,36] by chemical techniques, and Song, Ten Haaf, and Li [25,37,38] obtained needle-, block-, bar-, and rod-shaped ones by vacuum techniques. Salavati-Niasari and Zhang et al. [20,39] synthesized urchin- and dandelion-like microstructures by using aqueous chemical methods containing TGA and/or thioacetamide. These researchers conducted a comprehensive analysis to propose that the TGA plays a crucial role in the development of such Bi_2_S_3_ microstructures. 

The observations above indicate that the morphological development of nanostructured Bi_2_S_3_ appears to be unaffected by the synthesis method and is primarily determined by the crystal structure of Bi_2_S_3_.

Our results significantly differ from others previously reported. In our study, nanosized Bi_2_S_3_ particles self-assembled into worm-like nanostructures almost at the onset of precipitation. Subsequently, the self-assembly steps of these 1D nanostructures produced sponge-, urchin-, dandelion-, or flower-like Bi_2_S_3_ microstructures. The dandelion formation was favored when the synthesis was carried out at room temperature, regardless of the TGA content. Whereas, for the synthesis at 70 °C, all the prepared samples exhibited a similar morphology as that developed by the BiS10X series, resembling a flower-like structure. On the basis of these observations, the hierarchical self-assembly pathway leading to the formation of bismuth sulfide microstructures is primarily dictated by the crystalline structure of Bi_2_S_3_ and by the chemistry of the solution in which the digestion process takes place. Notice that digestion was carried out at room temperature. 

In our experiments, the role of TGA as an assembly director agent was not clear enough. In this sense, our interpretation differs from that of Salavati et al. [16], who proposed that TGA directs the final microstructure shape of Bi_2_S_3_. Instead, our results suggest that the crystal habit of Bi_2_S_3_, the synthesis temperature, and the solution digestion are the key factors influencing the microstructure formation. By understanding these factors, we can gain valuable insights into the synthesis of bismuth sulfide microstructures and potentially control their shape and properties.

Overall, this research demonstrates the fascinating hierarchical self-assembly process that leads to the formation of a great variety of Bi_2_S_3_ microstructures using simple and cheap synthesis techniques. The study also highlights the impact of digestion on the refinement of these structures, providing valuable insights into their formation and development. The SEM analysis has provided valuable information related to the potential applications of Bi_2_S_3_ in various fields such as optoelectronics, catalysis, and energy storage. The thorough investigation of the morphology of Bi_2_S_3_ powders by SEM has enhanced our understanding of their physical properties and paved the way for their utilization in advanced device research.

## 4. Conclusions

In conclusion, this study introduces a simple and cost-effective method for producing Bi_2_S_3_ microstructures in a variety of sizes and shapes. The synthesis process involves co-precipitation at low temperatures (25 and 70 °C), followed by a 24 h digestion period. This method results in a highly pure Bi_2_S_3_ powder. The unique morphology of the Bi_2_S_3_ microstructures is achieved through a hierarchical self-assembly process and a digestion mechanism.

Starting with Bi_2_S_3_ particles ranging from 15 to 40 nm in size, it was observed that these nanoparticles self-assemble into worm-like 1D nanostructures during the precipitation reaction. These nanoworms then further assemble into sponge-, urchin-, and dandelion or flower-like Bi_2_S_3_ microstructures. What sets our results apart from previous studies is the role of worm-like nanostructures as building blocks for more complex forms. These structures undergo shape transformations due to Ostwald ripening, nanocrystal adhesion, and reshaping during the digestion process.

The final shape of the Bi_2_S_3_ microstructures, particularly the crystal habit of Bi_2_S_3_, is influenced by the chemical composition of the solution and temperature. Dandelion-like and flower-like microstructures were observed at 25 and 70 °C, respectively. The resulting Bi2S3 microstructures morphology have a high surface area, making them ideal for applications in sensing and catalysis.

## Data Availability

Data are contained within the article.
